# Supplementary material for: Modeling glioblastoma heterogeneity as a dynamic network of cell states
Source: Mol Syst Biol. 2021 Sep 16;17(9):e10105. doi: 10.15252/msb.202010105 (PMC8444284; doi:10.15252/msb.202010105)
Supplement: Supplementary file 6 — Source Data for Figure 5 [file MSB-17-e10105-s004.zip › Figure5A_sourcedata/GSEA_3017/nosigdb_stateA.GseaPreranked.1622795026485/RICHARDS_NOWAKOWSKI_SCIENCE_2017_MGE_IPC1_UPREG.html]

Details for gene set RICHARDS\_NOWAKOWSKI\_SCIENCE\_2017\_MGE\_IPC1\_UPREG[GSEA]

|  || Dataset | state53017 |
| Phenotype | NoPhenotypeAvailable |
| Upregulated in class | na\_pos |
| GeneSet | RICHARDS\_NOWAKOWSKI\_SCIENCE\_2017\_MGE\_IPC1\_UPREG |
| Enrichment Score (ES) | 0.3965221 |
| Normalized Enrichment Score (NES) | 3.3253975 |
| Nominal p-value | 0.0 |
| FDR q-value | 0.0 |
| FWER p-Value | 0.0 |
Table: GSEA Results Summary

  

Fig 1: Enrichment plot: RICHARDS\_NOWAKOWSKI\_SCIENCE\_2017\_MGE\_IPC1\_UPREG      
 Profile of the Running ES Score & Positions of GeneSet Members on the Rank Ordered List

  

| PROBE | GENE SYMBOL | GENE\_TITLE | RANK IN GENE LIST | RANK METRIC SCORE | RUNNING ES | CORE ENRICHMENT || 1 | CDK1 |  |  | 3 | 1.006 | 0.0119 | Yes |
| 2 | MKI67 |  |  | 4 | 0.926 | 0.0261 | Yes |
| 3 | KIF11 |  |  | 6 | 0.870 | 0.0383 | Yes |
| 4 | UBE2C |  |  | 7 | 0.793 | 0.0505 | Yes |
| 5 | KIF20B |  |  | 10 | 0.749 | 0.0597 | Yes |
| 6 | CEP55 |  |  | 11 | 0.747 | 0.0712 | Yes |
| 7 | BUB3 |  |  | 12 | 0.740 | 0.0826 | Yes |
| 8 | KIF5B |  |  | 16 | 0.687 | 0.0895 | Yes |
| 9 | KPNA2 |  |  | 20 | 0.667 | 0.0962 | Yes |
| 10 | HIST1H4C |  |  | 21 | 0.659 | 0.1063 | Yes |
| 11 | ASPM |  |  | 27 | 0.639 | 0.1101 | Yes |
| 12 | PIF1 |  |  | 30 | 0.627 | 0.1174 | Yes |
| 13 | CCNB1 |  |  | 37 | 0.599 | 0.1193 | Yes |
| 14 | CKS2 |  |  | 40 | 0.587 | 0.1259 | Yes |
| 15 | HMMR |  |  | 43 | 0.559 | 0.1321 | Yes |
| 16 | NCAPG |  |  | 44 | 0.556 | 0.1407 | Yes |
| 17 | HJURP |  |  | 47 | 0.539 | 0.1466 | Yes |
| 18 | GDI2 |  |  | 49 | 0.537 | 0.1536 | Yes |
| 19 | CKS1B |  |  | 50 | 0.530 | 0.1618 | Yes |
| 20 | DBF4 |  |  | 55 | 0.514 | 0.1649 | Yes |
| 21 | RPL22L1 |  |  | 56 | 0.512 | 0.1728 | Yes |
| 22 | SMC3 |  |  | 58 | 0.504 | 0.1793 | Yes |
| 23 | KIF14 |  |  | 59 | 0.503 | 0.1871 | Yes |
| 24 | CENPE |  |  | 62 | 0.496 | 0.1923 | Yes |
| 25 | MIS18BP1 |  |  | 64 | 0.495 | 0.1987 | Yes |
| 26 | CCNA2 |  |  | 65 | 0.495 | 0.2063 | Yes |
| 27 | TPX2 |  |  | 66 | 0.488 | 0.2138 | Yes |
| 28 | DLGAP5 |  |  | 76 | 0.475 | 0.2102 | Yes |
| 29 | HIST1H1B |  |  | 77 | 0.475 | 0.2176 | Yes |
| 30 | AURKA |  |  | 78 | 0.474 | 0.2249 | Yes |
| 31 | CDC20 |  |  | 80 | 0.472 | 0.2309 | Yes |
| 32 | VRK1 |  |  | 82 | 0.470 | 0.2370 | Yes |
| 33 | UBE2T |  |  | 83 | 0.470 | 0.2442 | Yes |
| 34 | PLK1 |  |  | 89 | 0.464 | 0.2453 | Yes |
| 35 | TUBB4B |  |  | 93 | 0.462 | 0.2488 | Yes |
| 36 | CENPF |  |  | 94 | 0.461 | 0.2559 | Yes |
| 37 | TOP2A |  |  | 95 | 0.457 | 0.2629 | Yes |
| 38 | HIST1H1D |  |  | 98 | 0.456 | 0.2675 | Yes |
| 39 | ZWINT |  |  | 106 | 0.447 | 0.2659 | Yes |
| 40 | TGIF1 |  |  | 107 | 0.447 | 0.2728 | Yes |
| 41 | AURKB |  |  | 108 | 0.446 | 0.2797 | Yes |
| 42 | ZEB1 |  |  | 109 | 0.446 | 0.2865 | Yes |
| 43 | SEPHS1 |  |  | 114 | 0.437 | 0.2884 | Yes |
| 44 | CDCA8 |  |  | 115 | 0.437 | 0.2951 | Yes |
| 45 | KIF23 |  |  | 116 | 0.436 | 0.3019 | Yes |
| 46 | DEPDC1 |  |  | 119 | 0.432 | 0.3061 | Yes |
| 47 | MELK |  |  | 123 | 0.427 | 0.3090 | Yes |
| 48 | BUB1B |  |  | 129 | 0.421 | 0.3094 | Yes |
| 49 | RTKN2 |  |  | 141 | 0.413 | 0.3025 | Yes |
| 50 | GTSE1 |  |  | 146 | 0.411 | 0.3039 | Yes |
| 51 | CCNB2 |  |  | 147 | 0.411 | 0.3103 | Yes |
| 52 | ANLN |  |  | 152 | 0.407 | 0.3117 | Yes |
| 53 | MAD2L1 |  |  | 153 | 0.406 | 0.3180 | Yes |
| 54 | PBX3 |  |  | 157 | 0.404 | 0.3206 | Yes |
| 55 | KIFC1 |  |  | 160 | 0.402 | 0.3243 | Yes |
| 56 | ARHGAP11A |  |  | 162 | 0.399 | 0.3293 | Yes |
| 57 | KIF2C |  |  | 165 | 0.397 | 0.3330 | Yes |
| 58 | CCAR1 |  |  | 170 | 0.395 | 0.3342 | Yes |
| 59 | NDC80 |  |  | 173 | 0.393 | 0.3378 | Yes |
| 60 | FAM83D |  |  | 180 | 0.389 | 0.3365 | Yes |
| 61 | ARHGAP19 |  |  | 187 | 0.385 | 0.3352 | Yes |
| 62 | BUB1 |  |  | 188 | 0.384 | 0.3411 | Yes |
| 63 | KIF22 |  |  | 197 | 0.377 | 0.3372 | Yes |
| 64 | CDCA3 |  |  | 203 | 0.372 | 0.3369 | Yes |
| 65 | PTMA |  |  | 206 | 0.371 | 0.3402 | Yes |
| 66 | SMC4 |  |  | 217 | 0.367 | 0.3337 | Yes |
| 67 | RAB13 |  |  | 220 | 0.366 | 0.3369 | Yes |
| 68 | SPC25 |  |  | 226 | 0.363 | 0.3364 | Yes |
| 69 | INCENP |  |  | 227 | 0.362 | 0.3420 | Yes |
| 70 | NCAPH |  |  | 232 | 0.359 | 0.3427 | Yes |
| 71 | CENPA |  |  | 243 | 0.352 | 0.3360 | Yes |
| 72 | H2AFZ |  |  | 245 | 0.351 | 0.3402 | Yes |
| 73 | TTK |  |  | 262 | 0.340 | 0.3260 | Yes |
| 74 | KNSTRN |  |  | 263 | 0.340 | 0.3313 | Yes |
| 75 | TUBB |  |  | 264 | 0.340 | 0.3365 | Yes |
| 76 | CENPJ |  |  | 270 | 0.339 | 0.3356 | Yes |
| 77 | LRR1 |  |  | 273 | 0.337 | 0.3384 | Yes |
| 78 | DEPDC1B |  |  | 275 | 0.335 | 0.3424 | Yes |
| 79 | ESCO2 |  |  | 282 | 0.333 | 0.3402 | Yes |
| 80 | ORC6 |  |  | 283 | 0.333 | 0.3453 | Yes |
| 81 | NPM1 |  |  | 292 | 0.328 | 0.3407 | Yes |
| 82 | C21orf58 |  |  | 294 | 0.328 | 0.3445 | Yes |
| 83 | C2orf69 |  |  | 297 | 0.326 | 0.3471 | Yes |
| 84 | G2E3 |  |  | 302 | 0.325 | 0.3473 | Yes |
| 85 | MND1 |  |  | 304 | 0.324 | 0.3511 | Yes |
| 86 | RPS6 |  |  | 306 | 0.324 | 0.3548 | Yes |
| 87 | BRCA2 |  |  | 308 | 0.323 | 0.3586 | Yes |
| 88 | CENPN |  |  | 310 | 0.323 | 0.3624 | Yes |
| 89 | MLLT10 |  |  | 311 | 0.322 | 0.3673 | Yes |
| 90 | SFRP2 |  |  | 320 | 0.319 | 0.3626 | Yes |
| 91 | DNA2 |  |  | 321 | 0.318 | 0.3675 | Yes |
| 92 | TUBA1C |  |  | 322 | 0.318 | 0.3724 | Yes |
| 93 | TNFAIP8L1 |  |  | 336 | 0.314 | 0.3614 | Yes |
| 94 | CDCA5 |  |  | 341 | 0.312 | 0.3614 | Yes |
| 95 | CLIC1 |  |  | 342 | 0.311 | 0.3662 | Yes |
| 96 | CHD7 |  |  | 343 | 0.311 | 0.3710 | Yes |
| 97 | PRC1 |  |  | 350 | 0.310 | 0.3684 | Yes |
| 98 | RPS3 |  |  | 356 | 0.307 | 0.3671 | Yes |
| 99 | ANP32B |  |  | 357 | 0.307 | 0.3718 | Yes |
| 100 | ECT2 |  |  | 365 | 0.305 | 0.3680 | Yes |
| 101 | CCNF |  |  | 366 | 0.305 | 0.3727 | Yes |
| 102 | TTF2 |  |  | 367 | 0.305 | 0.3774 | Yes |
| 103 | CDCA2 |  |  | 369 | 0.304 | 0.3809 | Yes |
| 104 | MYEF2 |  |  | 371 | 0.303 | 0.3844 | Yes |
| 105 | NET1 |  |  | 378 | 0.301 | 0.3817 | Yes |
| 106 | CKAP2 |  |  | 379 | 0.301 | 0.3863 | Yes |
| 107 | RRM2 |  |  | 383 | 0.300 | 0.3873 | Yes |
| 108 | CENPW |  |  | 386 | 0.299 | 0.3895 | Yes |
| 109 | TEX30 |  |  | 388 | 0.298 | 0.3929 | Yes |
| 110 | PHF19 |  |  | 393 | 0.297 | 0.3926 | Yes |
| 111 | RACGAP1 |  |  | 399 | 0.293 | 0.3911 | Yes |
| 112 | HMGB1 |  |  | 403 | 0.292 | 0.3919 | Yes |
| 113 | CCT5 |  |  | 409 | 0.290 | 0.3903 | Yes |
| 114 | DCP2 |  |  | 416 | 0.289 | 0.3875 | Yes |
| 115 | HMGB2 |  |  | 424 | 0.288 | 0.3834 | Yes |
| 116 | NUSAP1 |  |  | 430 | 0.286 | 0.3818 | Yes |
| 117 | NUF2 |  |  | 434 | 0.285 | 0.3825 | Yes |
| 118 | MIS18A |  |  | 439 | 0.284 | 0.3820 | Yes |
| 119 | NCAPD2 |  |  | 440 | 0.283 | 0.3864 | Yes |
| 120 | LMNB1 |  |  | 442 | 0.282 | 0.3895 | Yes |
| 121 | CDC25C |  |  | 443 | 0.282 | 0.3939 | Yes |
| 122 | KIF20A |  |  | 447 | 0.281 | 0.3946 | Yes |
| 123 | ANP32E |  |  | 453 | 0.280 | 0.3928 | Yes |
| 124 | BCHE |  |  | 456 | 0.280 | 0.3947 | Yes |
| 125 | MNS1 |  |  | 464 | 0.278 | 0.3905 | Yes |
| 126 | ATAD2 |  |  | 470 | 0.275 | 0.3887 | Yes |
| 127 | EZH2 |  |  | 471 | 0.275 | 0.3929 | Yes |
| 128 | SKA1 |  |  | 475 | 0.274 | 0.3935 | Yes |
| 129 | SHCBP1 |  |  | 477 | 0.274 | 0.3965 | Yes |
| 130 | KIF4A |  |  | 504 | 0.269 | 0.3691 | No |
| 131 | UBE2S |  |  | 507 | 0.269 | 0.3708 | No |
| 132 | HAUS8 |  |  | 518 | 0.267 | 0.3628 | No |
| 133 | RCC1 |  |  | 528 | 0.265 | 0.3560 | No |
| 134 | SKA3 |  |  | 531 | 0.264 | 0.3576 | No |
| 135 | ZFP36L1 |  |  | 532 | 0.264 | 0.3617 | No |
| 136 | KIF15 |  |  | 538 | 0.263 | 0.3597 | No |
| 137 | CDKN3 |  |  | 539 | 0.263 | 0.3637 | No |
| 138 | STIL |  |  | 545 | 0.261 | 0.3617 | No |
| 139 | CENPO |  |  | 549 | 0.260 | 0.3620 | No |
| 140 | CCDC77 |  |  | 553 | 0.259 | 0.3624 | No |
| 141 | FBXO5 |  |  | 557 | 0.258 | 0.3627 | No |
| 142 | SPDL1 |  |  | 560 | 0.258 | 0.3643 | No |
| 143 | DDX39A |  |  | 562 | 0.258 | 0.3670 | No |
| 144 | EIF5A |  |  | 583 | 0.253 | 0.3467 | No |
| 145 | TMPO |  |  | 584 | 0.253 | 0.3506 | No |
| 146 | NES |  |  | 603 | -0.252 | 0.3326 | No |
| 147 | CD99 |  |  | 604 | -0.252 | 0.3365 | No |
| 148 | NLGN4X |  |  | 613 | -0.259 | 0.3308 | No |
| 149 | ABAT |  |  | 617 | -0.259 | 0.3311 | No |
| 150 | FAM111A |  |  | 618 | -0.259 | 0.3351 | No |
| 151 | SORT1 |  |  | 664 | -0.279 | 0.2848 | No |
| 152 | DCTN3 |  |  | 665 | -0.280 | 0.2891 | No |
| 153 | RAB3IP |  |  | 667 | -0.280 | 0.2922 | No |
| 154 | CKB |  |  | 676 | -0.287 | 0.2869 | No |
| 155 | RPS27L |  |  | 688 | -0.295 | 0.2781 | No |
| 156 | PON2 |  |  | 743 | -0.338 | 0.2178 | No |
| 157 | MSI2 |  |  | 771 | -0.361 | 0.1906 | No |
| 158 | EPHA4 |  |  | 778 | -0.367 | 0.1890 | No |
| 159 | CXXC5 |  |  | 809 | -0.391 | 0.1586 | No |
| 160 | TMSB4X |  |  | 850 | -0.443 | 0.1169 | No |
| 161 | TFDP2 |  |  | 858 | -0.448 | 0.1153 | No |
| 162 | FRMD4B |  |  | 925 | -0.626 | 0.0448 | No |
| 163 | ZBTB20 |  |  | 964 | -0.895 | 0.0125 | No |
| 164 | CDK6 |  |  | 966 | -0.920 | 0.0255 | No |
Table: GSEA details [plain text format]

  

Fig 2: RICHARDS\_NOWAKOWSKI\_SCIENCE\_2017\_MGE\_IPC1\_UPREG: Random ES distribution      
 Gene set null distribution of ES for **RICHARDS\_NOWAKOWSKI\_SCIENCE\_2017\_MGE\_IPC1\_UPREG**
